# Supplementary figures and images for: Characterization of increased mucus production of HT29-MTX-E12 cells grown under Semi-Wet interface with Mechanical Stimulation
Source: PLoS One. 2021 Dec 20;16(12):e0261191. doi: 10.1371/journal.pone.0261191 (PMC8687553; doi:10.1371/journal.pone.0261191)

Supporting figure 1

A

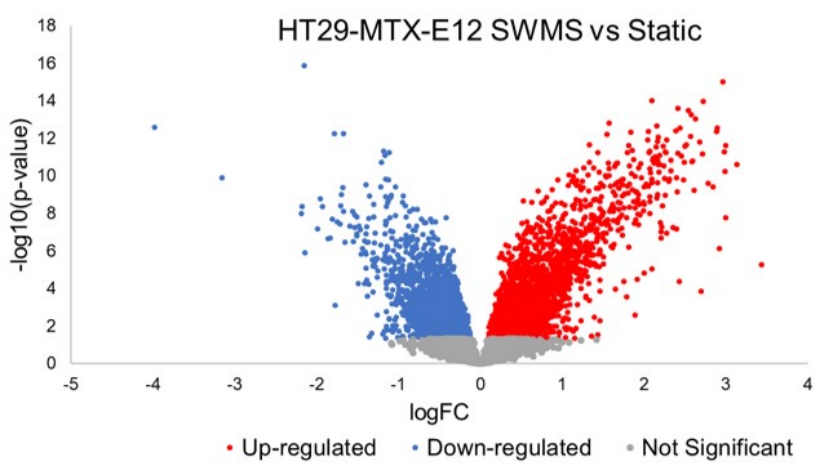

B

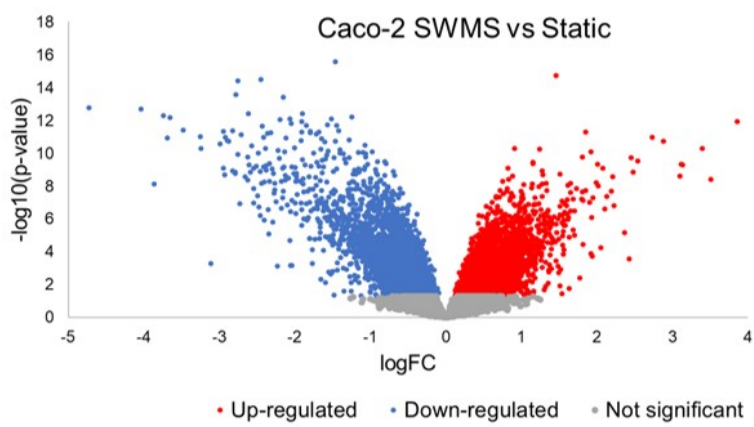

Supplement: S1 Fig — A) Volcano plot highlighting the Log Fold Change (logFC) on the x-axis and the corresponding p-values (-log(10)) on the y-axis for the comparison SWMS versus static conditions in HT29-MTX-E12 cells and B) Caco-2 cells. n = 3. (PDF) [file pone.0261191.s001.pdf]

Supporting Figure 5

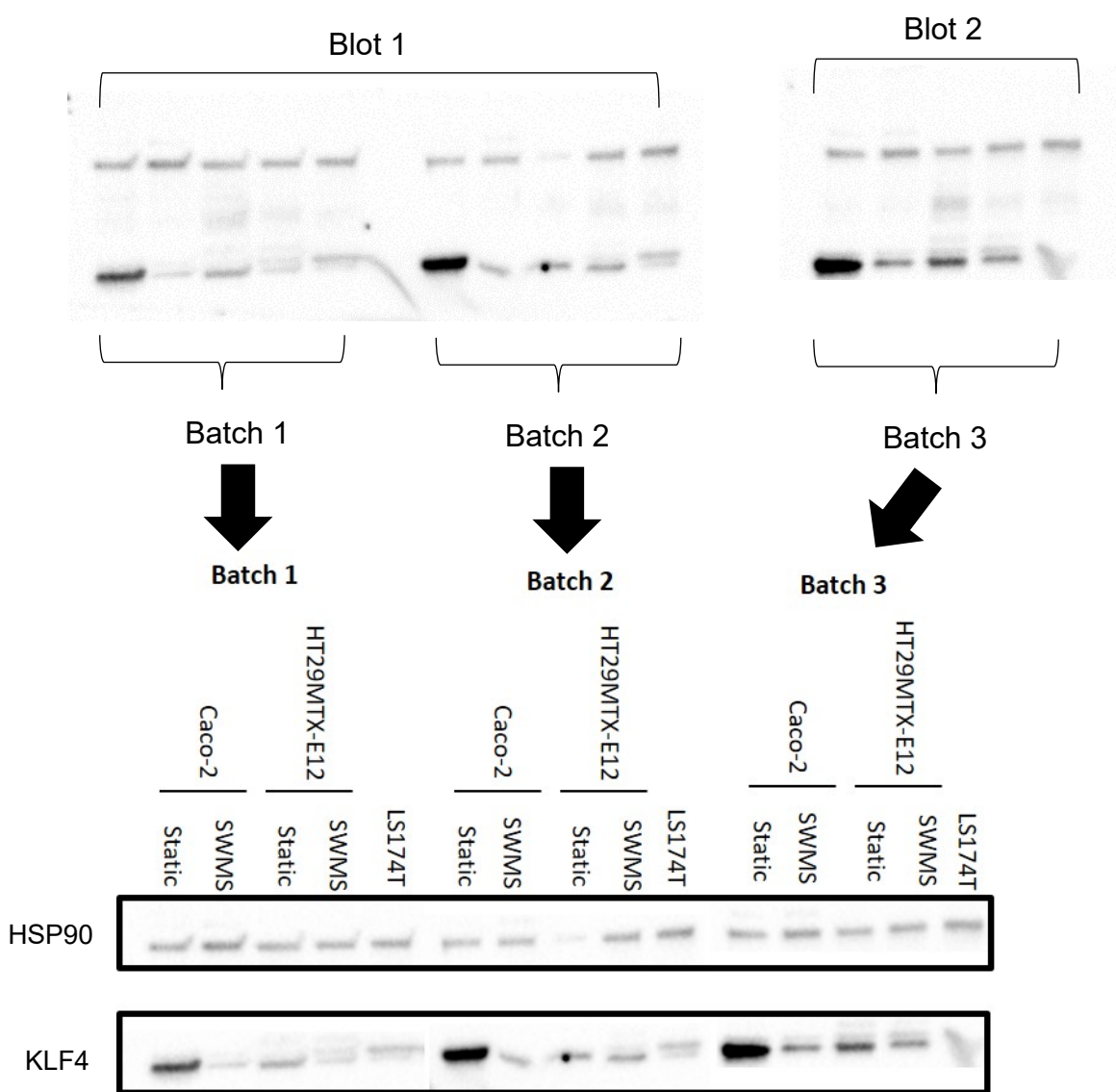

Supplement: S5 Fig — HSP90 was used as house-keeping protein. (PDF) [file pone.0261191.s005.pdf]

Supporting Figure 6

A

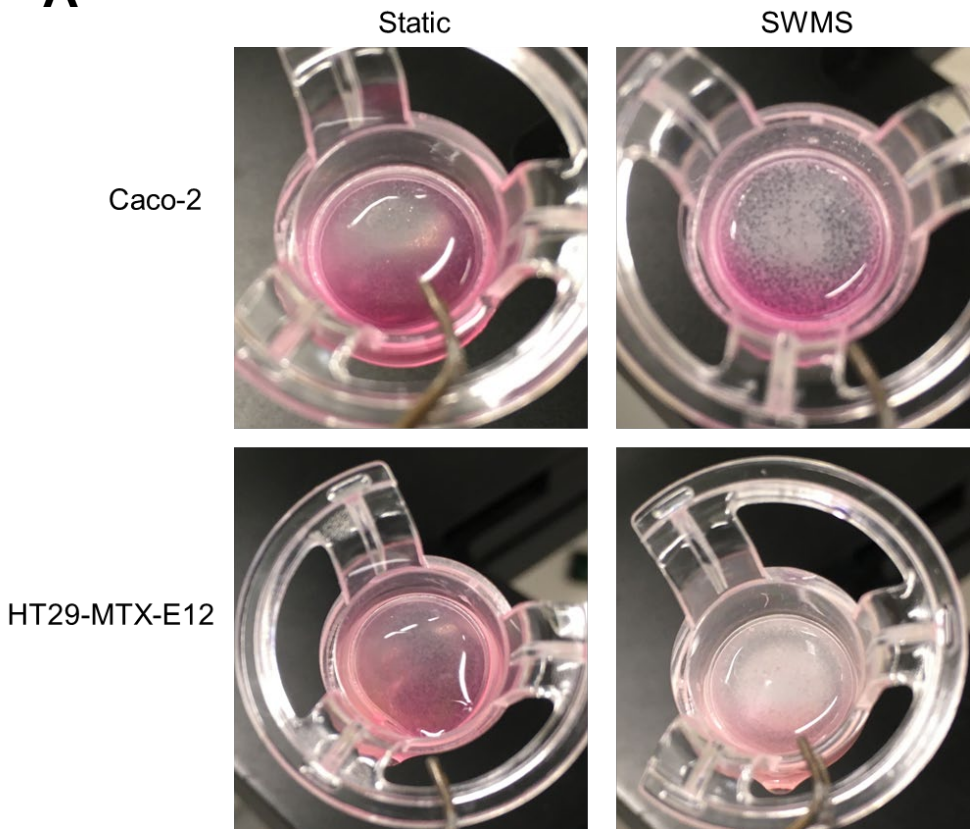

B

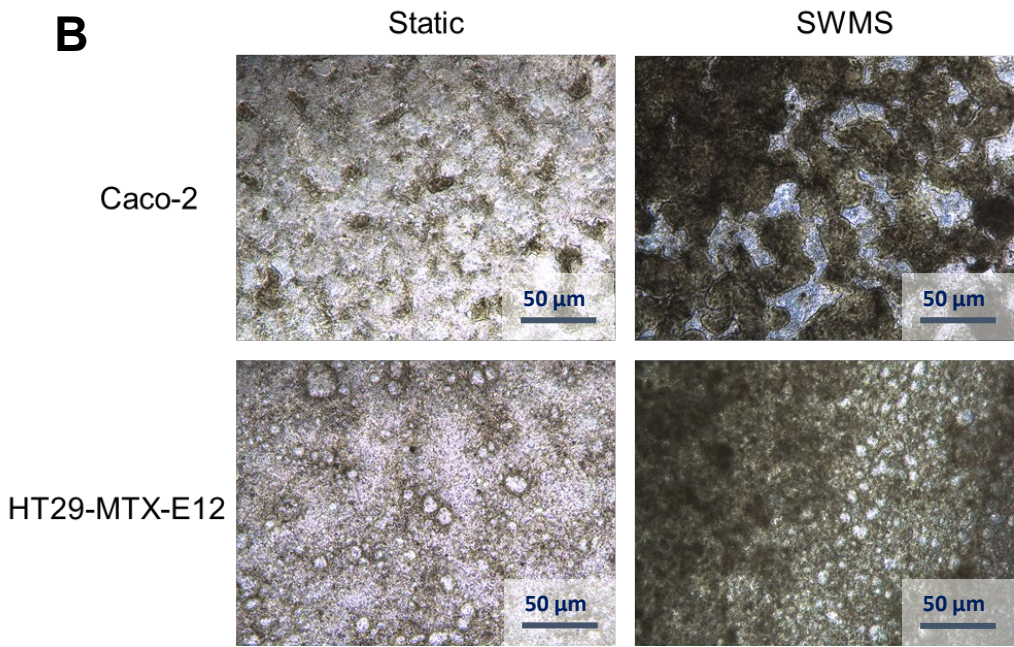

Supplement: S6 Fig — A) Pictures of HT29-MTX-E12 and Caco-2 cells grown under static and SWMS conditions at t = 15 days. B) Bright-field microscopy pictures (20x) of HT29-MTX-E12 and Caco-2 cells grown under static and SWMS condition, focussed on the centre of the Transwell membranes. (PDF) [file pone.0261191.s006.pdf]

Supporting Figure 7

A

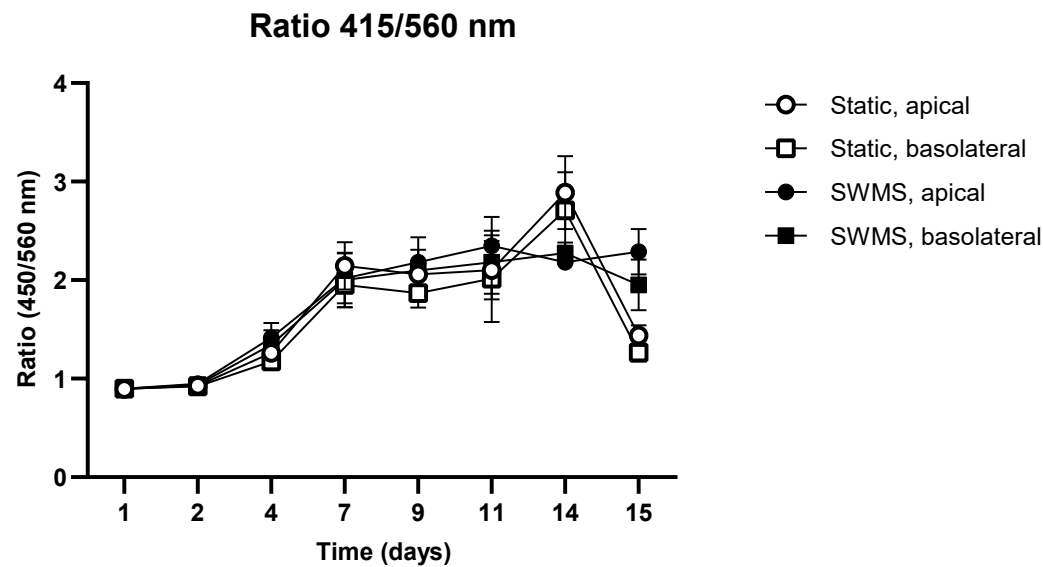

B

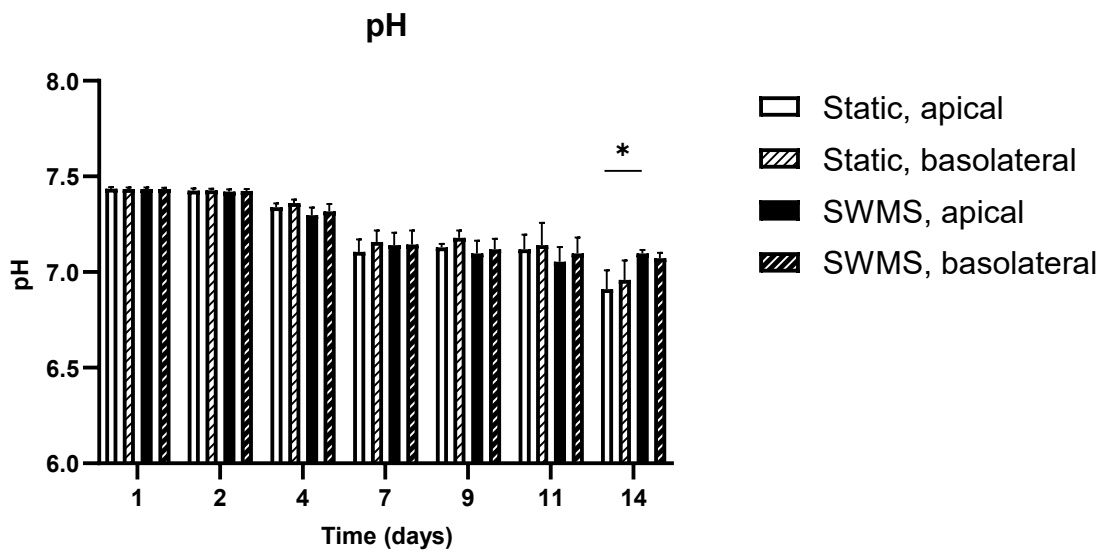

Supporting Figure 7 (cont.)

C

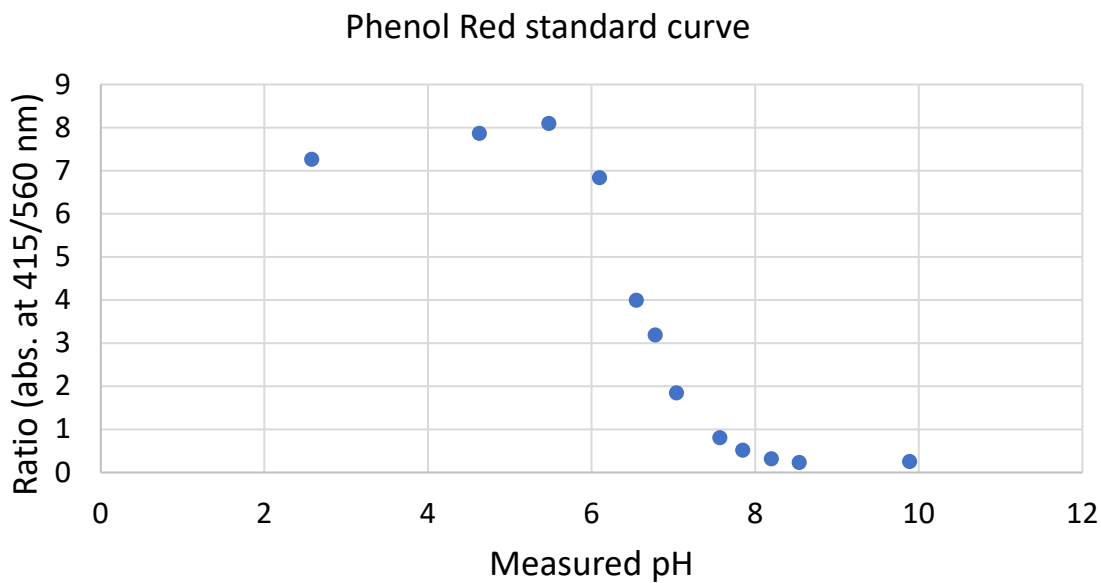

D

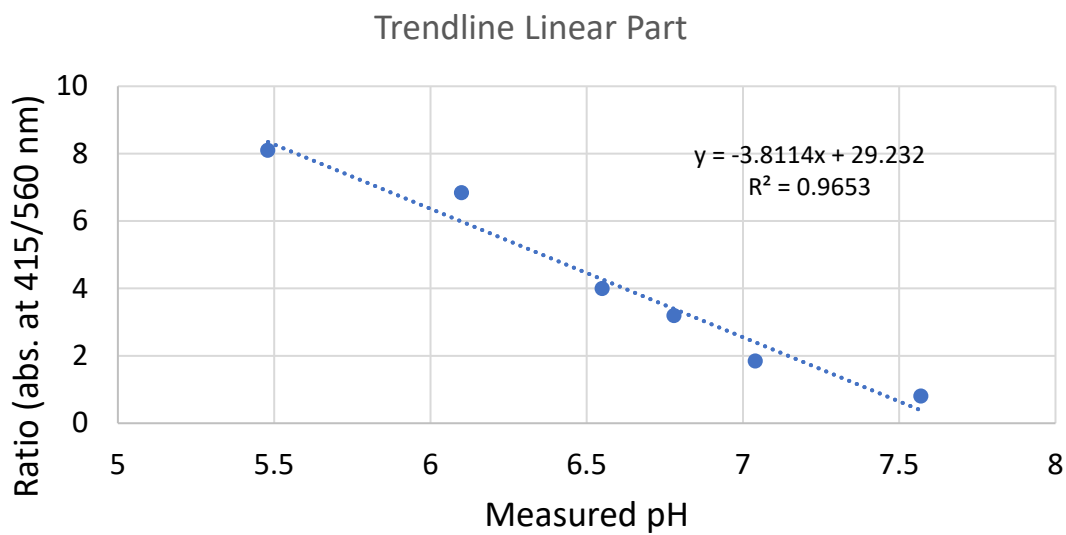

Supplement: S7 Fig — A) Ratio of Absorbance of cell culture medium (415 and 560 nm) of HT29-MTX-E12 and Caco-2 cells under static and SWMS conditions measured at 5% CO2. B) Medium pH of apical and basolateral compartments of HT29-MTX-E12 cells grown under static and SWMS conditions at t = 1–14 days. * p < 0.05; ** p < 0.01, n = 3 C) Standard curve of pH values and absorbance values of cell culture medium measured at 415/560 nm at 5% CO2. D) The linear part the standard curve, including trendline. (PDF) [file pone.0261191.s007.pdf]

Supporting Figure 8

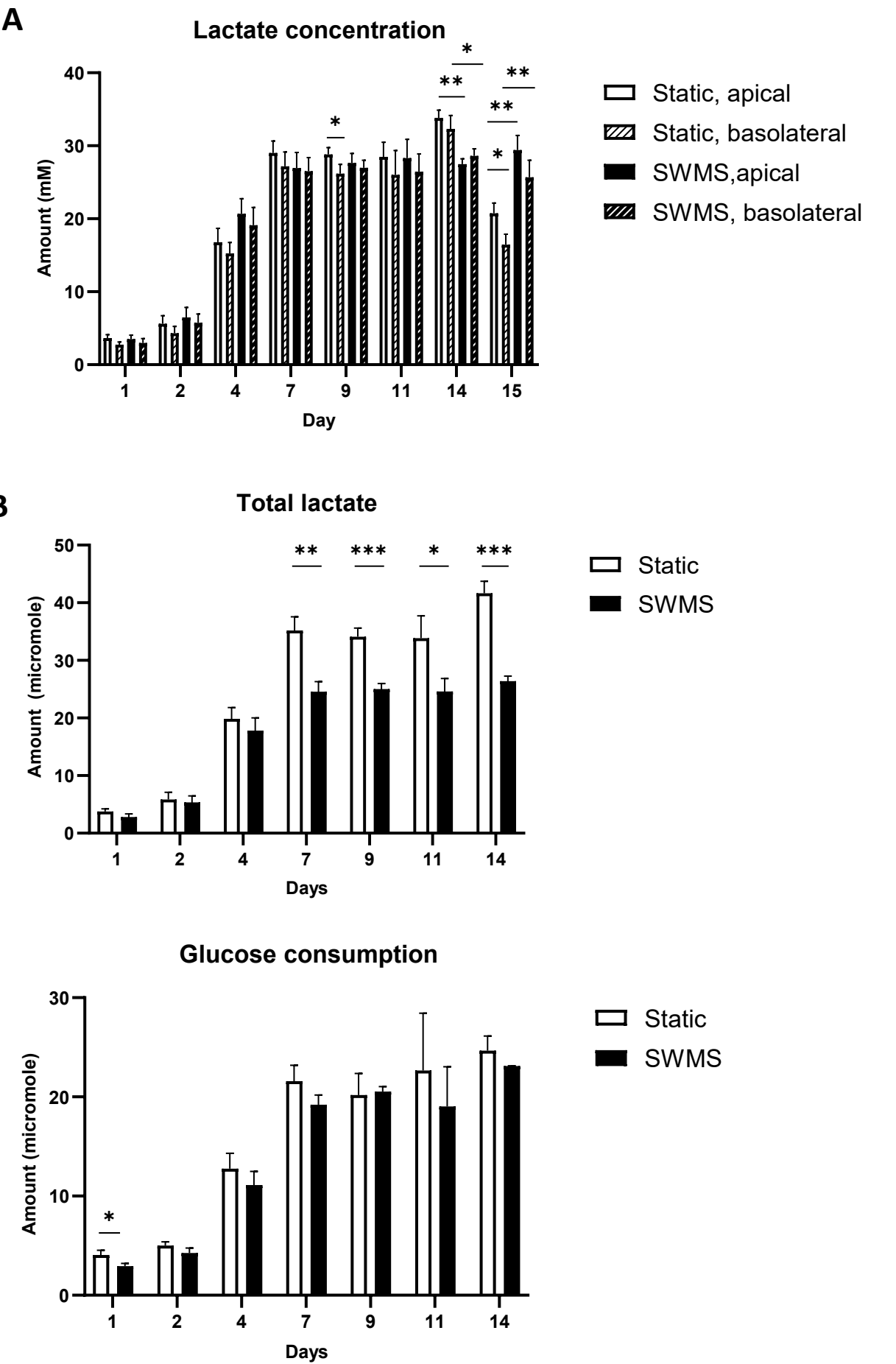

Supplement: S8 Fig — A) Lactate concentration (mM) in cell culture medium of HT29-MTX-E12 cells collected during every medium refreshing moment. B) Total lactate (micromole) produced per well in medium collected from apical and basolateral compartments of HT29-MTX-E12 grown under static or SWMS conditions, at t = 1–14 days. C) Total glucose (micromole) consumed per well from medium collected from apical and basolateral compartments of HT29-MTX-E12 grown under static and SWMS conditions, at t = 1–14 days. (PDF) [file pone.0261191.s008.pdf]
