# Supplementary material for: Characterization of increased mucus production of HT29-MTX-E12 cells grown under Semi-Wet interface with Mechanical Stimulation
Source: PLoS One. 2021 Dec 20;16(12):e0261191. doi: 10.1371/journal.pone.0261191 (PMC8687553; doi:10.1371/journal.pone.0261191)

Supporting Figure 2

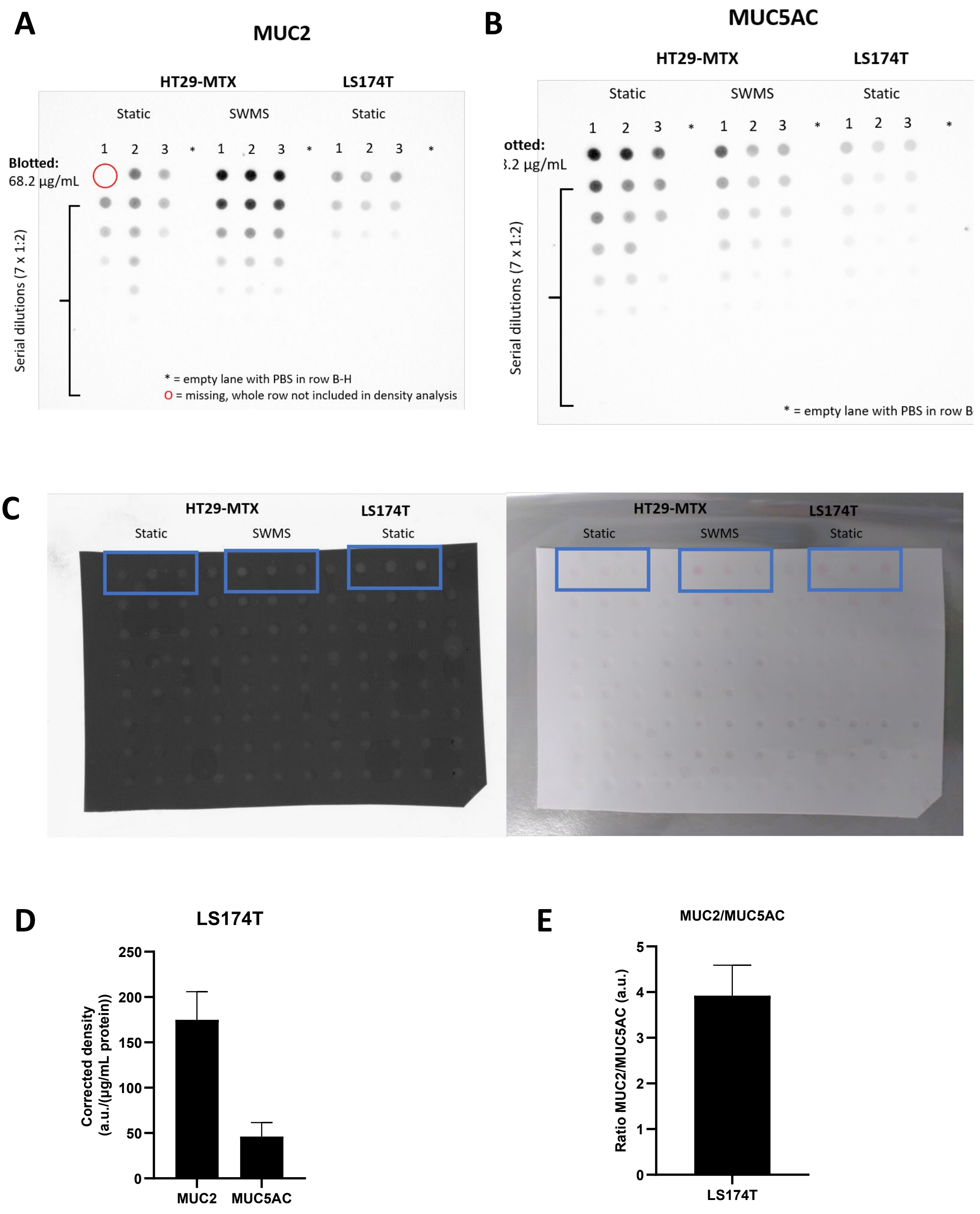

Supporting Figure 2

F

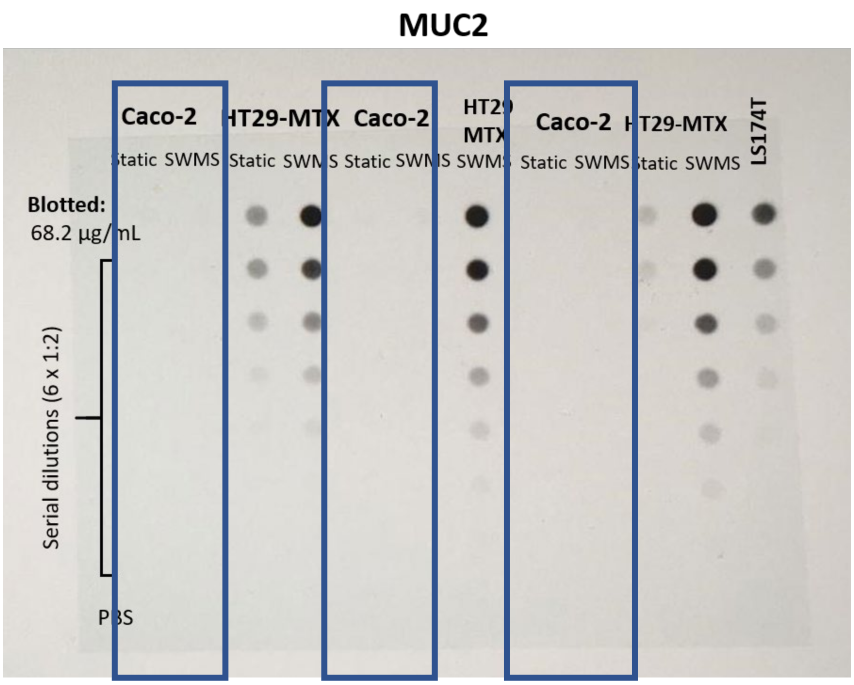

G

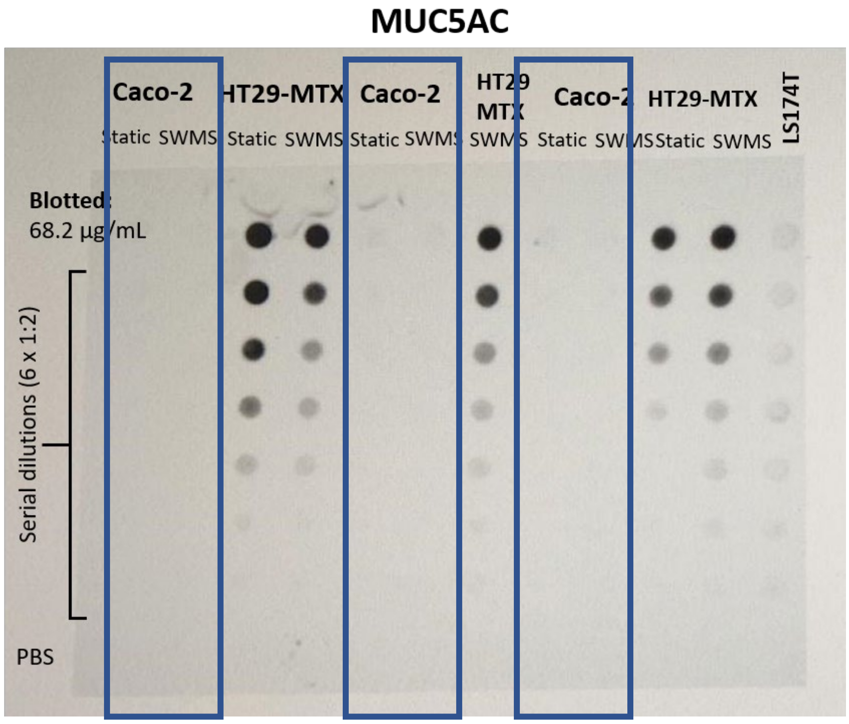

Supplement: S2 Fig — Overview of Dot Blot results for A) MUC2 and B) MUC5AC in HT29-MTX-E12 cells cultured under static and SWMS conditions and LS174T cells. A concentration of 68.2 μg/mL was blotted and seven times serially diluted 1:2. In column 4, 8 and 12, PBS was used as a negative control. C) Images of Ponceau Red staining (colorimetric and photographic) that were used as reference for total protein content. Protein density was based on the colorimetric image. D) Protein expression of MUC2 and MUC5AC in LS174T cells, expressed as density (a.u.) per ug/mL protein blotted, after correction of Ponceau Red density (n = 3) E) Ratio of MUC2 and MUC5AC protein expression in LS174T cells (n = 3). Original Dot Blots for Caco-2 are displayed in F) for MUC2 and G) for MUC5AC. Caco-2 data are indicated with a blue box. HT29-MTX-E12 and LS174T samples have been repeated including additional replicates for figure A and B. (PDF) [file pone.0261191.s002.pdf]
