# Supplementary material for: Characterization of increased mucus production of HT29-MTX-E12 cells grown under Semi-Wet interface with Mechanical Stimulation
Source: PLoS One. 2021 Dec 20;16(12):e0261191. doi: 10.1371/journal.pone.0261191 (PMC8687553; doi:10.1371/journal.pone.0261191)
Supplement: S3 Fig — The top 20 up- and downregulated genes shared between HT29-MTX-E12 and Caco-2 cells is given in the tables. (PDF) [file pone.0261191.s003.pdf]

Supporting Figure 3

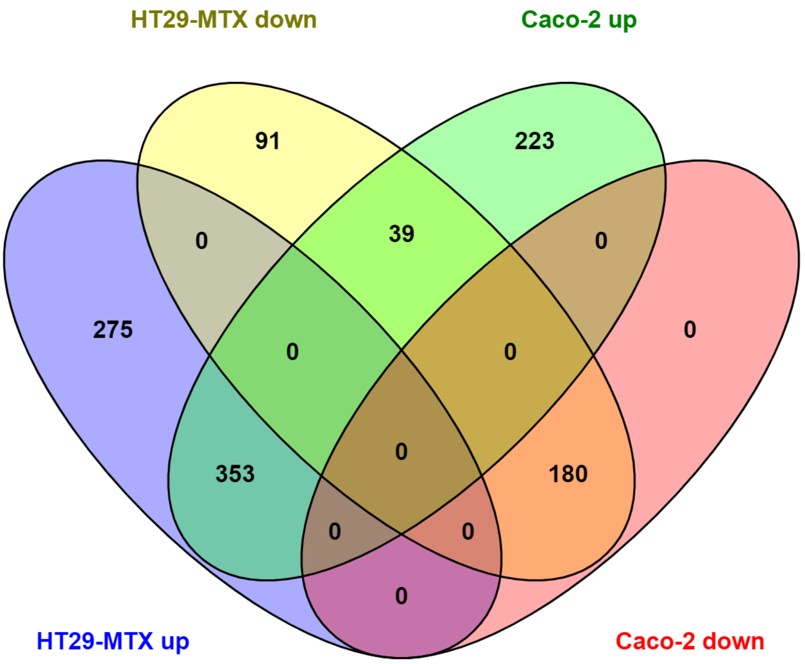

Up = FC ≥ 1.5
Down = FC ≤ -1.5

Top 20 upregulated genes shared between HT29-MTX and Caco-2

Gene name	FC in HT29-MTX	p-value	FC in Caco-2	p-value	Gene function
RNY4P23	10.80	0.00	2.12	0.05	RNY4 pseudogene 23
KIF14	8.00	0.00	1.52	0.01	kinesin family member 14
KIF20A	7.99	0.00	1.76	0.01	kinesin family member 20A
DLGAP5	7.91	0.00	2.04	0.00	DLG associated protein 5
HMMR	7.82	0.00	1.52	0.00	hyaluronan mediated motility receptor
H4C1	7.56	0.00	2.86	0.00	H4 clustered histone 1
SPC25	7.41	0.00	2.06	0.00	SPC25 component of NDC80 kinetochore complex
H3C8	7.20	0.00	2.17	0.00	H3 clustered histone 8
TOP2A	6.89	0.00	1.79	0.00	DNA topoisomerase II alpha
CCNB1	6.59	0.00	1.75	0.00	cyclin B1
CENPF	6.57	0.00	1.82	0.00	centromere protein F
NDC80	6.42	0.00	1.90	0.00	NDC80 kinetochore complex component
H1-5	6.04	0.00	2.57	0.00	H1.5 linker histone, cluster member
ASPM	6.02	0.00	2.02	0.00	abnormal spindle microtubule assembly
PRR11	5.97	0.00	1.52	0.00	proline rich 11
CENPE	5.95	0.00	1.88	0.00	centromere protein E
NUSAP1	5.82	0.00	1.72	0.00	nucleolar and spindle associated protein 1
FOXM1	5.72	0.00	1.81	0.00	forkhead box M1

Supporting Figure 3 (cont.)

Top 20 downregulated genes shared between HT29-MTX and Caco-2

Gene name	FC in HT29-MTX	p-value	FC in Caco-2	p-value	Gene function
SLC6A10P	-1.51	0.01	-2.29	0.00	solute carrier family 6 member 10, pseudogene
CD55	-1.51	0.00	-2.11	0.00	CD55 molecule (Cromer blood group)
HLA-C	-1.51	0.00	-2.07	0.00	major histocompatibility complex, class I, C
SLC46A3	-1.51	0.00	-1.97	0.00	solute carrier family 46 member 3
NRAD1	-1.51	0.00	-1.64	0.00	non-coding RNA in the aldehyde dehydrogenase 1A pathway
BCAT1	-1.52	0.00	-2.60	0.00	branched chain amino acid transaminase 1
ELF3-AS1	-1.52	0.00	-1.58	0.00	ELF3 antisense RNA 1
ARL14	-1.52	0.01	-2.08	0.00	ADP ribosylation factor like GTPase 14
MIR4268	-1.52	0.01	-1.50	0.03	microRNA 4268
MUC13	-1.52	0.00	-3.20	0.00	mucin 13, cell surface associated
ZNF625	-1.52	0.04	-1.66	0.03	zinc finger protein 625
VSIR	-1.53	0.00	-1.71	0.00	V-set immunoregulatory receptor
UACA	-1.53	0.00	-1.53	0.00	uveal autoantigen with coiled-coil domains and ankyrin repeats
TSPAN18	-1.53	0.00	-3.25	0.00	tetraspanin 18
EGFL7	-1.53	0.00	-1.57	0.00	EGF like domain multiple 7
PHKA2-AS1	-1.53	0.01	-2.82	0.00	PHKA2 antisense RNA 1
EPB41L1	-1.54	0.00	-1.89	0.00	erythrocyte membrane protein band 4.1 like 1
PTPRH	-1.54	0.00	-1.60	0.00	protein tyrosine phosphatase receptor type H
PDK1	-1.55	0.00	-2.79	0.00	pyruvate dehydrogenase kinase 1
LRP1	-1.55	0.00	-1.66	0.00	LDL receptor related protein 1
