# Supplementary material for: Characterization of increased mucus production of HT29-MTX-E12 cells grown under Semi-Wet interface with Mechanical Stimulation
Source: PLoS One. 2021 Dec 20;16(12):e0261191. doi: 10.1371/journal.pone.0261191 (PMC8687553; doi:10.1371/journal.pone.0261191)
Supplement: S1 Raw images — (PDF) [file pone.0261191.s011.pdf]

Raw files of Supporting Figure 2 of which data was used to generate bar charts of Figure 2B and C

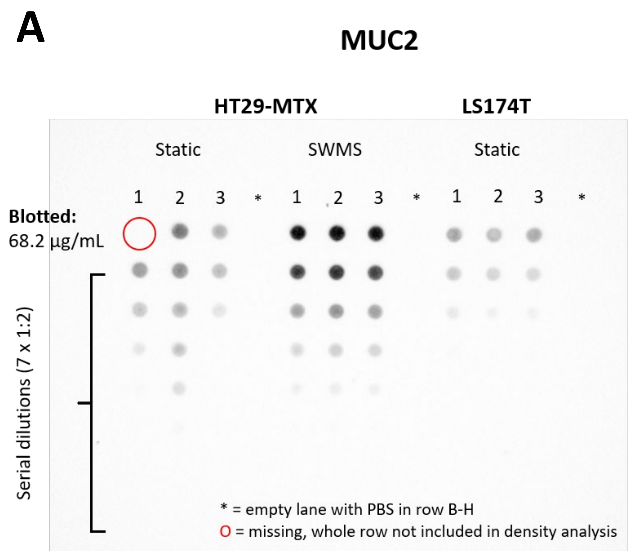

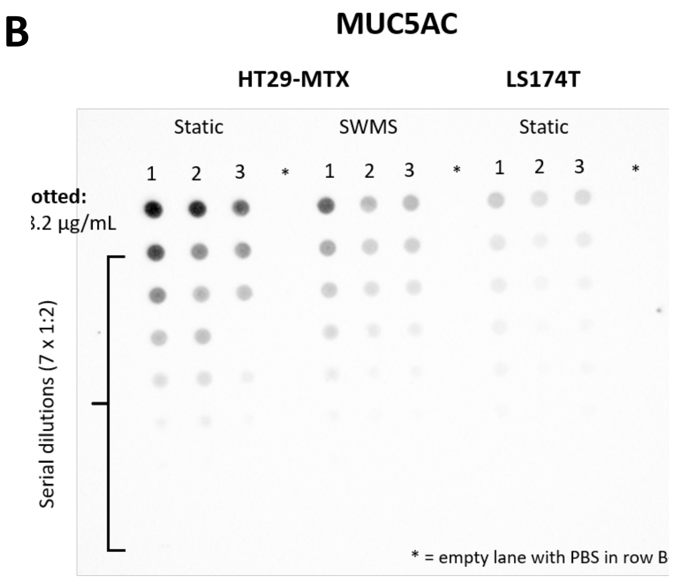

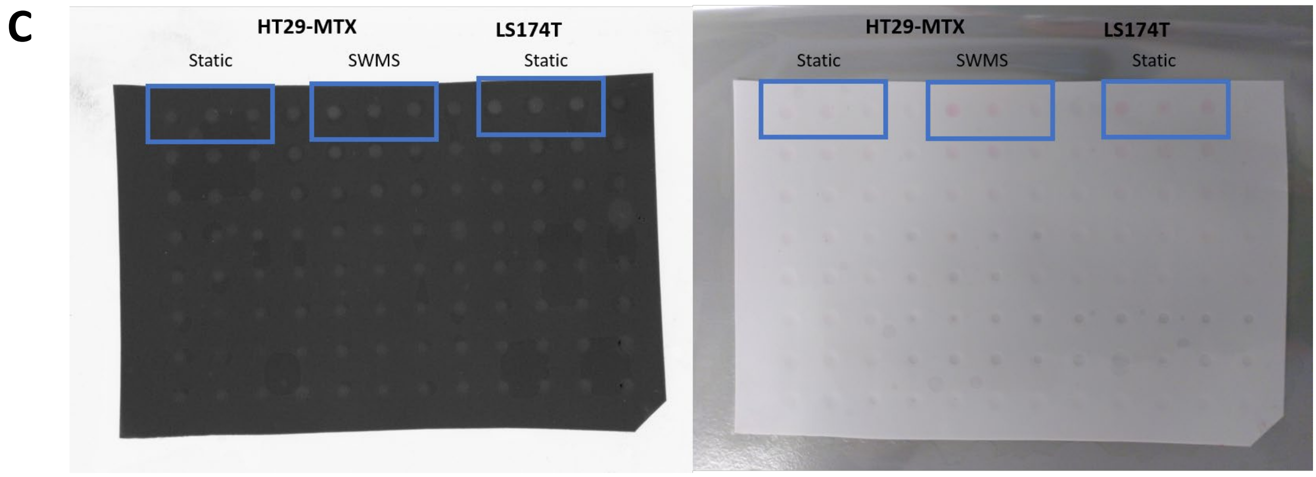

Signals were quantified using the ChemiDoc MP system (Bio-Rad) and Clarity ECL substrate (Bio-Rad)

Raw files of Supporting Figure 2 supporting claim that Caco-2 did not show protein expression of MUC2 and MUC5AC

F

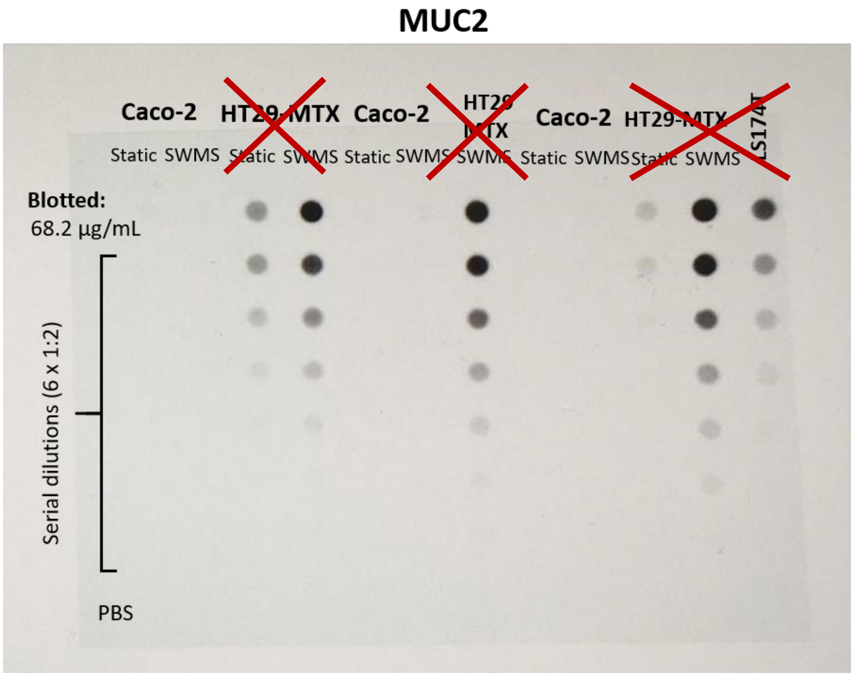

G

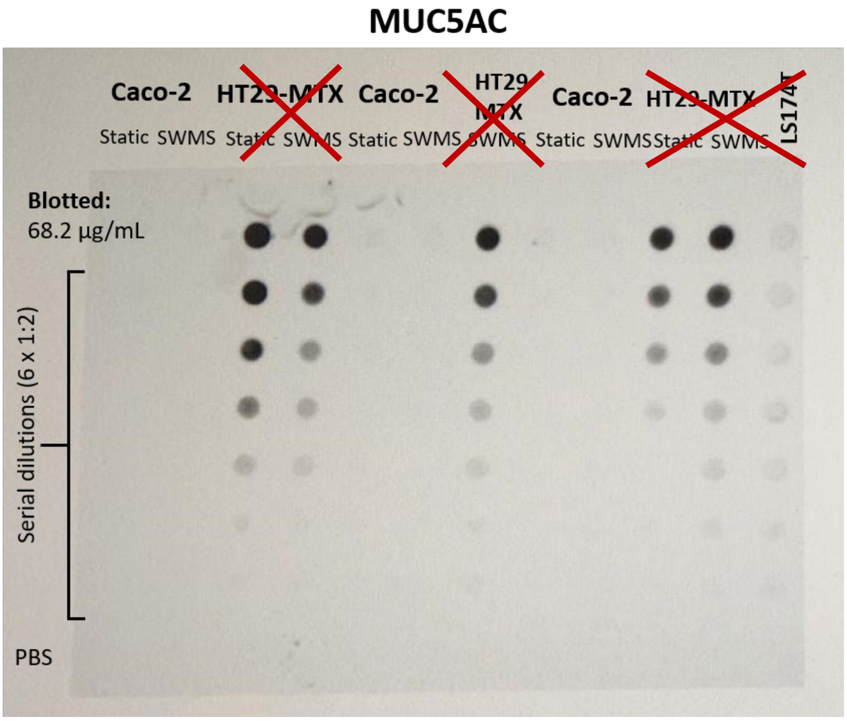

Signals were quantified using the ChemiDoc MP system (Bio-Rad) and Clarity ECL substrate (Bio-Rad)

Raw files of Supporting Figure 5 of which data was used to generate figure 3A and B

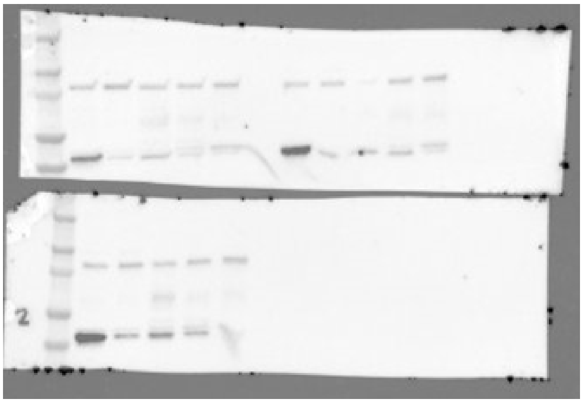

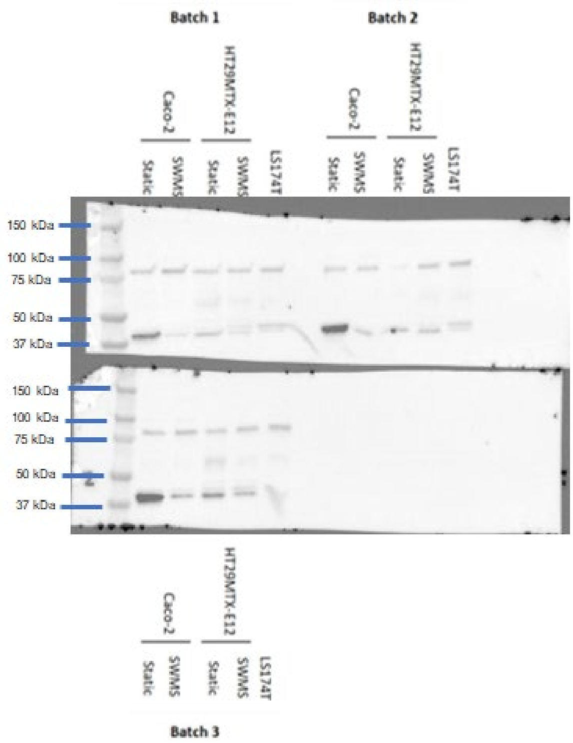

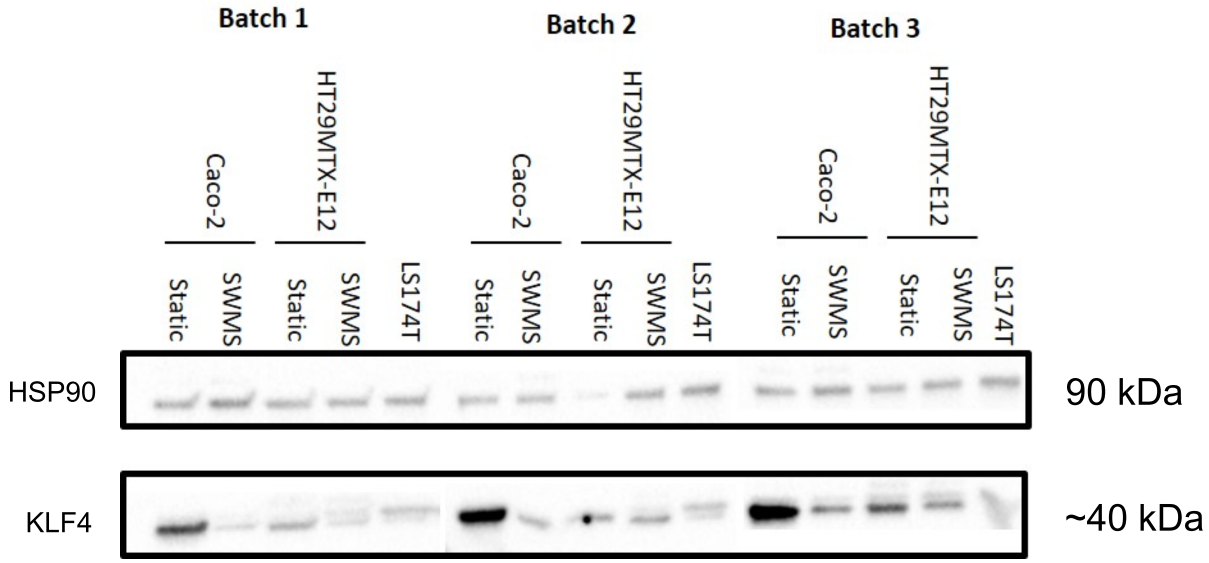

Signals were quantified using the ChemiDoc MP system (Bio-Rad) and Clarity ECL substrate (Bio-Rad)
