# Supplementary material for: Characterization of increased mucus production of HT29-MTX-E12 cells grown under Semi-Wet interface with Mechanical Stimulation
Source: PLoS One. 2021 Dec 20;16(12):e0261191. doi: 10.1371/journal.pone.0261191 (PMC8687553; doi:10.1371/journal.pone.0261191)
Supplement: Attachment — Submitted filename: Reponse to reviewers.docx [file pone.0261191.s012.docx]

Dear Prof. dr. Dudeja and reviewers,

Thank you for reviewing our manuscript ‘Characterization of increased mucus production of HT29-MTX-E12 cells grown under Semi-Wet interface with Mechanical Stimulation’ (PONE-D-21-14708). We highly appreciate your input and we incorporated your comments, which definitely improved the quality of our manuscript.

In brief, as requested, we included extra biological replicates for the quantitation of MUC2 and MUC5AC protein expression and performed FITC-dextran experiments to complement the TEER data. Additionally, we further strengthened and/or clarified the rationale and discussion of the model that we characterized. Finally, we adapted the manuscript to the required manuscript formatting as requested for PLOS One.

Please find our point-by-point to the reviewers’ comments below. We are willing to respond to any further questions and comments you may have regarding our revised manuscript.

Kind regards, also on behalf of the co-authors,

Janneke Elzinga and Benthe van der Lugt

PS All original uncropped and unadjusted images underlying our blot results can now be found in the Supporting information (S2 and S5 Figure).

------------------------------------------------------------------------------------------------------------------------

The reviewers’ original comments are displayed in bold, our comments are displayed in italics.

*Line numbers refer to the manuscript with tracked changes.*

**Reviewer #1: This is an interesting study looking at the differential gene expressions in intestinal epithelial cells in static and semi-wet interface with mechanical stimulation (SWMS) culture conditions, particularly in terms of mucus production. The manuscript is written very well and the results are presented appropriately. I have only minor comments.**

*** Please include the relevance and purpose of mechanical stimulation in the introduction.**

Thank you for pointing this out. The use of mechanical stimulation (MS) to obtain increased MUC2 production, is adapted from Navabi *et al.* 2013. Unfortunately, the authors of this paper did not explain the rationale behind applying MS, but a few more recent studies do describe the positive effect of mechanical stimulation on MUC2 production in Caco-2 cells and organoid models. In line 90-91, we refer to the results of a study by Kim *et al.,* 2012. This paper describes the first gut-on-chip and claims that the physiological shear stress in the gut is 0.009 dyne/cm^2^. Therefore, it seems a relevant parameter to include in an *in vitro* model. To further strengthen the application of mechanical stimulation on mucus production in HT29-MTX-E12 cells in particular, we refer in lines 655-658 to the findings of a recently obtained preprint in our revised manuscript (Lindner, preprint).

*** It is not clear if the results represent more than one experiment.**

We understand that our statement on the number of replicates was not very clear. The phrase “Unless stated otherwise, all experiments were performed in triplicate.” has been replaced by “All experiments were performed in three biological replicates” (line 241) or – as was the case for TEER and FITC-dextran experiments: “For each biological replicate (= independent experiment), two technical replicates (= wells) were measured” (lines 156-158 and lines 176-177). Upon request, additional replicates of the Dot Blot of HT29-MTX-E12 under static conditions and LS174T cells have been included in the revised manuscript in S2 Figure.

*** The quality of Fig.4 is not helpful for the readers. Please replace them. The authors may consider adding a simple cellular stain such as crystal violet or a nuclear dye for better visualization.**

Thank you for this valid point. Because this figure did not really contribute to the main conclusion, we moved it to the supplementary files. We included a scale bar upon request.

*** Please include SGLT1 (SLC5A1) in the discussion on glucose metabolism.**

Thank you for the suggestion. We did see a slightly and significantly higher expression of this gene under SWMS in HT29-MTX-E12 (FC = 1.43, p < 0.001) but a large and significant downregulation in Caco-2 (FC = -5.0, p < 1 · 10^-11^). As far as we know, though we found a considerably high RMA of ~7 in HT29-MTX-E12 under static and SWMS conditions, this transporter is known to be mainly expressed in the small-intestine *in vivo*. Instead, we decided to focus on the gene encoding GLUT1 (SLC2A1) with a RMA of ~11, as this protein has been studied and found differentially regulated in another *in vitro* model applying semi-wet interface (Klasvogt *et al.* 2017)

*** TEER studies can be supplemented with flux of paracellular probes such as FITC-dextran.**

In the revised manuscript, we incorporated data using FITC-dextran to quantify paracellular permeability of HT29-MTX-E12 under SWMS and static conditions (Figure 4C-D). Interestingly, our newly performed assays with FITC-dextran of both 4 kDa en 40 kDa demonstrate that in presence of a mucus layer, cells grown under SWMS demonstrate a significantly higher permeability then under static conditions. After washing off the mucus with N-Acetyl-L-Cysteine, we did not observe any differences in paracellular permeability, which complements our TEER data. It is possible that FITC-dextran may not be the right probe to study permeability of the mucus layer, a.o. because of its hydrophilic properties, which makes it hard to draw conclusions on the permeability of the mucus layer in our newly performed experiments. In earlier stages of our research, we did an attempt to study mucus permeability using fluorescence beads, but this required a very advanced set-up, and we, unfortunately, do not have all required facilities in our lab.

*** There is scope for shortening the discussion; it is lengthy in the present form.**

Thank you for pointing this out. We did an attempt to further shorten the discussion, by sticking to the available evidence as much as possible.

*** Considering the manuscript’s focus on characterizing the mechanisms of mucin production, it is worth demonstrating the mucus layer morphologically/ e.g. by immunohistochemistry in static versus SWMS cultures.**

Indeed, we acknowledge the added value of morphological evidence of the established mucus layer. We did several attempts to visualize the mucus layer, but have been struggling with preserving the mucus layer during fixation. This obstacle seems to be commonly known and has also been mentioned by Navabi *et al.*, 2013. They, however, did manage to visualize the thicker adherent mucus layer in HT29-MTX-E12 under SWMS versus static conditions and we would like to refer to that paper for visual, yet qualitative evidence. For our own study, we decided to stick with (semi-)quantitative evidence of the mucin produced on both gene and protein level.

*** I missed cytoskeleton-associated changes which might be important in view of mechano-stimulation.**

Thank you for pointing out this category of genes. Because they overlap partly with the cell-cycle related genes, which was among the most significantly enriched upregulated pathways, we refer to a separate panel of cytoskeleton-related in the results (lines 331-332). Additionally, we briefly mention the potential role of cytoskeleton-related as well as mechanosensitive genes in the discussion (lines 676-678), both for which we included additional gene panels in Supporting File 1. These panels are based on curated gene sets for the corresponding categories, but regulated genes from both panels will need further investigation in the context of the SWMS model.

**References mentioned above**

Navabi N, McGuckin MA, Lindén SK. Gastrointestinal cell lines form polarized epithelia with an adherent mucus layer when cultured in semi-wet interfaces with mechanical stimulation. PLoS One. 2013;8(7):e68761.

Kim HJ, Huh D, Hamilton G, Ingber DE. Human gut-on-a-chip inhabited by microbial flora that experiences intestinal peristalsis-like motions and flow. Lab Chip. 2012;12(12):2165-74.

Lindner *et al.*, preprint. https://doi.org/10.22541/au.160733083.37849435/v1

Klasvogt S, Zuschratter W, Schmidt A, Kröber A, Vorwerk S, Wolter R, *et al.* Air-liquid interface enhances oxidative phosphorylation in intestinal epithelial cell line IPEC-J2. Cell Death Discov. 2017;3:17001.

--------------------------------------------------------------------------------------------------------------------------------------

**Reviewer #2: The manuscript by Elzinga *et al.* adds on to previous knowledge regarding the mucus production of HT29-MTX-E12 cells grown under semi-wet interface with mechanical stimulation. Although the work provides insights into this model for *in vitro* culture to study mucus, the following needs to be addressed to improve the overall impact of the work.**

**Major concerns**

**1. The authors bring up a point that this model capture human intestinal (colonic) mucus *in vitro*, however, the levels of other MUCs were significantly low in this model. Does this occur *in vivo*? Then why do the authors think this will be physiologically relevant? Discuss and explain the drawbacks etc.**

Thank you for pointing this out. We should have made more clear that our focus has been on secreted mucins affected by the SWMS conditions, as demonstrated earlier by Navabi *et al.* (2013). In our revised manuscript, we tried to put more stress on this particular focus. Additionally, in the discussion, we discuss the potential drawbacks of the SWMS conditions, pointing at the low expression of *MUC4*, amongst others, and make suggestions to look further in the (equally important) transmembrane mucins 1 and 4 (lines 690-696).

**2. Figure 1 A, how about MUC1 and MUC4? Please include other MUC proteins as well. Why were dot blots conducted instead of standard western blot? There should be enough protein for this assay please explain why. Also include western blot results which is normalized to a loading control such as GAPDH and Actin HSP etc. with a representative blot and densitometric analysis with statistics conducted of at least n>4. Please include at least n=3 when plotting data (Figure 1B) in addition.**

Thank you for this suggestion. See our previous comment about why we did not look at protein expression of MUC1 and MUC4. Western Blot analysis of large molecules such as mucins is limited, as heavy glycosylation and multimerization limit the use of conventional SDS-PAGE polyacrylamide gel and standard protein ladders (Ramsey *et al.* 2016). We chose to do Dot Blot, because it is another commonly used technique used to (semi-)quantify mucins (Harrop *et al.*, 2012). To correct for discrepancies in blotting efficiency between samples, we decided to correct for whole protein by staining with Ponceau Red. A loading control (HSP90) has been included for each biological replicate in the Western Blot experiments to detect KLF4, which showed equal expression across samples. For all protein experiments, we performed three technical replicates per biological replicate (n = 3), but we only performed Dot Blot on one of the technical replicates, similar as we did for the micro-array analysis. As suggested, for our revised manuscript, we reperformed the experiments and included two additional biological replicates for LS174T (was n = 1, previously) and a third biological replicate of HT29-MTX-E12 cultured under static conditions.

**3. It would be nice if the authors could supplement Figure 3 with FITC dextran data in addition to the TEER data.**

In the revised manuscript, we incorporated data using FITC-dextran to quantify paracellular permeability of HT29-MTX-E12 under SWMS and static conditions. We would like to refer to the further explanation of this experiments in the fifth question of reviewer 1. Copied from above: In the revised manuscript, we incorporated data using FITC-dextran to quantify paracellular permeability of HT29-MTX-E12 under SWMS and static conditions (Figure 4C-D). Interestingly, our newly performed assays with FITC-dextran of both 4 kDa en 40 kDa demonstrate that in presence of a mucus layer, cells grown under SWMS demonstrate a significantly higher permeability then under static conditions. After washing off the mucus with N-Acetyl-L-Cysteine, we did not observe any differences in paracellular permeability, which complements our TEER data. It is possible that FITC-dextran may not be the right probe to study permeability of the mucus layer, a.o. because of its hydrophilic properties, which makes it hard to draw conclusions on the permeability of the mucus layer in our newly performed experiments. In earlier stages of our research, we did an attempt to study mucus permeability using fluorescence beads, but this required a very advanced set-up, and we, unfortunately, do not have all required facilities in our lab.

**4. Figure 4, what do the authors need the reader to observe? The photograph of the trans-well membrane provides no information pertaining to the amount of mucus etc. Please use a staining to show the amount of MUC2 or goblet cell mucins by using PAS staining immunofluorescence etc. Also, always add a scalebar to all microscopy images.**

Thank you for this valid point. Because this figure did not really contribute to the main conclusion, we moved it to the supplementary files and included a scale bar upon request.

**5. It wasn’t clear, which was the mechanism behind increased MUC2 production in HT29-MTX-E12 cells grown under SWMS conditions? The authors have not speculated this aspect. For example. The transcription factors were unchanged however, there was an increase in IL-33 production. Could this be one of the mechanisms? Please add all this and improve the discussion section**

Thank you for pointing this out. In the discussion, we mention several mechanisms that could be at play, but our experiments are not conclusive on any of the proposed pathways. We also looked into the potential role of IL-33, given its high fold-upregulation at gene level in HT29-MTX-E12 under SWMS. In literature, however, we found that IL-33-induced increased expression of MUC2 is dependent on immune cells (Waddell *et al.*, 2019), which are not present in our model. As our transcriptome data also did not show regulation of other immune parameters and we did not look further explore immune parameters in our experiments, we decided not to include this hypothesis in our manuscript.

**6. Please summarize key findings of the paper in the final paragraph of the introduction section.**

Thank you for this suggestion. A short paragraph is included in the revised manuscript (lines 102-105).

**7. Please remove MUC2/MUC2 etc. from the graphical abstract.**

We do not entirely understand what you mean with this suggestion, but are willing to adapt the description of the genes and proteins in the figure, if anything is unclear.

**References mentioned above**

Navabi N, McGuckin MA, Lindén SK. Gastrointestinal cell lines form polarized epithelia with an adherent mucus layer when cultured in semi-wet interfaces with mechanical stimulation. PLoS One. 2013;8(7):e68761.

Ramsey KA, Rushton ZL, Ehre C. Mucin Agarose Gel Electrophoresis: Western Blotting for High-molecular-weight Glycoproteins. J Vis Exp. 2016;(112):54153.

Harrop CA, Thornton DJ, McGuckin MA. Detecting, visualising, and quantifying mucins. Methods Mol Biol. 2012;842:49-66.

Waddell A, Vallance JE, Hummel A, Alenghat T, Rosen MJ. IL-33 Induces Murine Intestinal Goblet Cell Differentiation Indirectly via Innate Lymphoid Cell IL-13 Secretion. J Immunol. 2019 Jan 15;202(2):598-607.

--------------------------------------------------------------------------------------------------------------------------------------

**Reviewer #3: In this article, Elzinga *et al.* examined the Semi-Wet interface with Mechanical Stimulation (SWMS) culture conditions with the commonly used HT29-MTX cells. This culture model was selected as it represents a simple, low cost method to examine intestinal mucus production. HT29-MTX cells grown in SWMS conditions exhibited elevated levels of MUC2, decreased levels of MUC5AC and MUC5B and alterations in several transmembrane mucins compared to cells cultured conventionally. These cells were enriched for cell cycle and DNA replication related pathways. Interestingly, NOTCH1 and ATOH1 were both significantly upregulated and TFFs (1,2, 3) were significantly downregulated by SWMS conditions. The authors also noted decreased pH and increased glucose consumption with SWMS conditions. With this work, the authors have provided mechanistic insights into mucus production in HT29-MTX cells using the SWMS culture system. Overall, the manuscript is well written and follows a logical flow. The conclusions are well supported by the findings and the discussion is appropriate. As a result, I have only minor comments.

1. Line 43: This is a very broad statement and adding more references in support of this claim would strengthen the introduction.**

We agree that this statement is very broad and that, especially “beneficial compounds”, may not be supported well enough. We changed the claim into: “…mucin-degrading bacterial species which liberate short-chain fatty acids for cross-feeding bacteria and the host (incl. refs)” (lines 46-47). And further emphasized the important role of mucin by adding: “As a nutrient source, mucin has been shown to be a major driver of intestinal microbiota composition *in vitro* (incl. ref)” (lines 48-49).

**2. Line 49: Paneth cells which produce lysozyme are found in the small intestine, but not normally in the colon (with the exception of IBD (PMID: 11851832). As result, the statement that colonic mucus contains lysozyme is misleading. Please remove lysozyme from the sentence.**

“Lysozyme” has been removed from this sentence.

**3. In Line 65, the authors state that organoids “are supposed to resemble the *in vivo* colonic mucosal layer more closely in terms of mucus composition”. The term “supposed to” seems vague; please remove “supposed to” since the MUC2 in organoids does resemble colonic mucus in terms of protein and glycosylation pattern.**

“Supposed to” has been removed from this sentence.

**4. Supplemental Figure 1 and 3 depict important findings and the manuscript would be improved if they were incorporated as main figures. Additionally Figure 4 seems more appropriate as a supplemental figure.**

Thank you for the suggestion. We incorporated part of your feedback, by moving part of Supplementary Figure 1 to the main text. We decided to keep Supplementary Figure 3 in the Supplementary Information, as our main conclusions are not based on the regulated genes shared by HT29-MTX-E12 and Caco-2 cells. Because Figure 4 also did not contribute to our main conclusions, we also moved it to the Supplementary Information, upon request.

**5. The changes in ion transporters is intriguing. NHE3 (SLC9A3) has been shown in mice to be the major regulator of intestine pH- was NHE3 altered in the HT29-MTX AI cells?**

Thank you for this suggestion. We included a single sentence on NHE3 in our results (lines 465-467), which was only expressed at low levels in HT29-MTX-E12 under both static and SWMS conditions (FC = -1.3, p < 0.05, RMA < 6).

**6. Were there any changes in other major goblet cell markers (AGR, CDX2, MEP1β, FCGBP, etc.)?**

Thank you for this suggestions. In the Supplementary information of our revised manuscript, we now included a whole panel of known (curated) goblet cell markers (Supplementary file 1). Apart from TFF3, as already mentioned in the manuscript, and PHGR1 (a recently characterized protein by Oltedal *et al.*, 2018), other major goblet cell markers were not differentially expressed under SWMS versus static conditions.

**7. Although the main focus of this paper is on a simple model of the intestinal epithelium (HT29-MTX cells), the discussion would benefit from inclusion of how SWMS conditions could be employed in organoid studies to create more robust models in this system as well.**

Thank you for this suggestion. To the best of our knowledge, SW and/or MS cultures have already been applied in organoids. Semi-wet conditions have recently been investigated in kidney organoids, amongst others (Gupta *et al.*, 2021). Mechanical stimulation has been described for brain-specific organoids, to increase oxygen supply (a.o. Lancaster and Knoblich, 2014). Furthermore, organoids have been grown on gut-on-chips, which already demonstrated the impact of flow on the mucus layer in these cells systems (Sontheimer Phelps *et al.*, 2020) Indeed, it would be interesting to speculate on the combined effect of SWMS in intestinal organoids, but as our discussion was already quite lengthy, we did not further elaborate on this.

**References mentioned above**

Oltedal S, Kørner H, Aasprong OG, *et al.* The Prognostic Relevance of Sentinel Lymph Node Metastases Assessed by PHGR1 mRNA Quantification in Stage I to III Colon Cancer. Transl Oncol. 2018;11(2):436-443.

Gupta AK, Ivancic DZ, Naved BA, Wertheim JA, Oxburgh L. An efficient method to generate kidney organoids at the air-liquid interface. J Biol Methods [Internet]. 2021Jun.29 [cited 2021Jul.27];8(2):e150.

Lancaster, M., Knoblich, J. Generation of cerebral organoids from human pluripotent stem cells. Nat Protoc 9, 2329–2340 (2014).

Sontheimer-Phelps A, Chou DB, Tovaglieri A, Ferrante TC, Duckworth T, Fadel C, *et al.* Human Colon-on-a-Chip Enables Continuous *In vitro* Analysis of Colon Mucus Layer Accumulation and Physiology. Cell Mol Gastroenterol Hepatol. 2020;9(3):507-26.
